# PU.1 and Haematopoietic Cell Fate: Dosage Matters

**DOI:** 10.1155/2011/808524

**Published:** 2011-08-10

**Authors:** Ka Sin Mak, Alister P. W. Funnell, Richard C. M. Pearson, Merlin Crossley

**Affiliations:** School of Biotechnology and Biomolecular Sciences, University of New South Wales, Sydney, NSW 2052, Australia

## Abstract

The ETS family transcription factor PU.1 is a key regulator of haematopoietic differentiation. Its expression is dynamically controlled throughout haematopoiesis in order to direct appropriate lineage specification. Elucidating the biological role of PU.1 has proved challenging. This paper will discuss how a range of experiments in cell lines and mutant and transgenic mouse models have enhanced our knowledge of the mechanisms by which PU.1 drives lineage-specific differentiation during haematopoiesis.

## 1. Introduction

Haematopoiesis is a lifelong process that generates the range of blood cell types that exhibit distinct and specialised functions. Transcription factors play a critical role in this complex and highly orchestrated process, directing multipotent haematopoietic stem cells (HSCs) towards lineage commitment by regulating lineage-specific gene expression, proliferation, and differentiation. The ETS family member PU.1 is one such transcription factor 

The PU.1 gene was first identified as a proviral integration site for the spleen focus forming virus (SFFV) in erythroleukaemias [1]. SFFV integration in the PU.1 locus leads to increased PU.1 transcription and subsequent erythroleukaemic transformation. It has since emerged that PU.1 is one of the major haematopoietic regulators, with a particular role in directing differentiation within the myeloid and lymphoid pathways [2]. Several PU.1 null and mutant mouse lines have been generated and exhibit varied phenotypes depending on the nature of PU.1 defect [3]. PU.1 knockout mice succumb to neonatal death and show a marked lack of myeloid cells, T and B cells [4, 5]. Erythropoiesis is also altered in the foetal livers of PU.1 null mice with erythroid progenitors displaying reduced self-renewal capacity and a propensity to differentiate prematurely [6]. 

**Figure 1:**
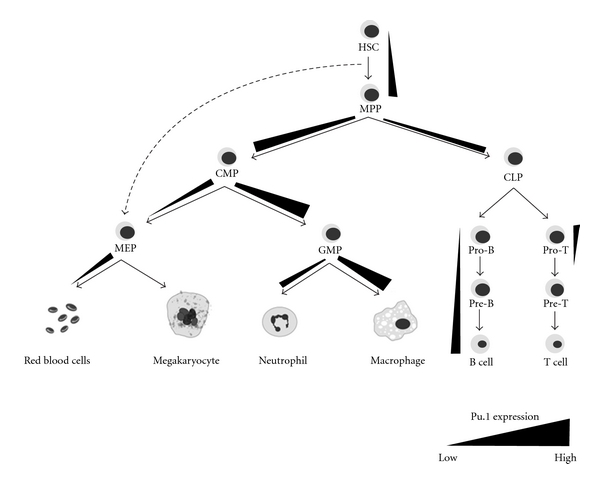
Schematic showing the changing expression of PU.1 during haematopoiesis. PU.1 levels, where known, are represented by gradient bars. Gradient bars are not drawn to scale. Differentiation pathways are denoted by arrows. Abbreviations: HSC, haematopoietic stem cell; MPP, multipotent progenitor; CMP, common myeloid progenitor; CLP, common lymphoid progenitor; MEP, megakaryocyte-erythroid progenitor; GMP, granulocyte-macrophage progenitor.

PU.1 is thus crucial in directing many facets of haematopoiesis and concordant with this, its expression fluctuates dynamically in the various haematopoietic differentiation pathways (Figure 1). Importantly, the regulation of differentiation by PU.1 is not merely via a “presence or absence of expression” mechanism but by a dose-dependent effect. For instance, the expression of PU.1 is low in long-term reconstituting (LT)-HSCs but rises as these progenitors become more lineage restricted and form precursor cells known as common myeloid progenitors (CMPs) and common lymphoid progenitors (CLPs). Upon further lineage differentiation and maturation, PU.1 is expressed at varied levels in mature blood cells, with higher levels found in macrophages than B cells and low levels in mature erythroid cells, megakaryocytes, and T cells [7–9]. Moreover, not only is PU.1 differentially expressed in the various haematopoietic cells, but also lineage specification is sensitive to, and directed by, the varied dosage of PU.1 in differentiating progenitor cells. In addition, inappropriate expression of PU.1 in specific haematopoietic cells can result in leukaemic transformation, as in the case of T-cell lymphomas and, as mentioned previously, erythroleukaemias [10, 11]. 

This paper focuses on how the expression pattern of PU.1 differs between different committed precursors and how this serves to determine cell fate. The interplay between PU.1 and other antagonistic haematopoietic regulators will also be discussed.

## 2. PU.1 Levels Are Important in Directing Haematopoietic Progenitor Cell Fate

PU.1 expression in HSCs is important for their self-renewal and for their development into CMPs and CLPs [12]. These two progenitor pools then further differentiate to form mature blood cells including megakaryocytes, red blood cells (RBCs), neutrophils and macrophages (all CMP derived), and B and T cells (CLP derived) (Figure 1). Iwasaki et al. showed that ablating PU.1 expression results in a decrease in HSC numbers by an order of magnitude and that CMPs, CLPs, and CMP progeny GMPs (granulocyte-monocyte progenitors; Figure 1) are all undetectable in PU.1 knockout foetal livers [12]. These haematopoietic defects are cell autonomous as PU.1 null HSCs fail to generate granular or myeloid colonies when cultured *in vitro* [12, 13]. Similarly, in competitive repopulation assays, PU.1 knockout foetal liver HSCs that were injected into lethally irradiated mice were barely detectable in peripheral blood, and the low numbers that were found within the bone marrow (BM) failed to generate monocytes and B and T lymphocytes [12, 14, 15]. Interestingly, the formation of megakaryocyte-erythroid progenitors (MEPs), however, remains intact in PU.1 knockout mice [12]. MEPs were conventionally thought to arise only from CMPs (Figure 1). However, the lack of CMPs and abundance of MEPs in PU.1 null mice suggest that MEPs may bypass the CMP stage and develop directly from HSCs, a hypothesis that is supported by several other studies [16–19]. Taken together, these data suggest that PU.1 is crucially important for normal development of HSCs into CMPs and CLPs but is dispensable for production of MEPs.

 In order to explore the role of PU.1 in haematopoietic lineages further, studies have been performed using reporter systems in which green fluorescent protein (GFP) has been knocked into the PU.1 locus to allow its expression pattern to be tracked throughout haematopoietic differentiation [6–8, 12]. While a low level of PU.1 is detected in LT-HSCs (Lin^−^ Sca-1^+^ c-kit^+^ CD34^−^), its expression increases as these cells become multipotent progenitors (MPPs; Lin^−^ Sca-1^+^ c-kit^+^ CD34^+^) and subsequently develop into both CMPs and CLPs [7, 12, 20]. At this stage, the level of PU.1 expression is decisive in permitting progression towards either the myeloid or lymphoid lineage [21, 22]. Culturing PU.1^+/−^ foetal liver progenitor cells (Lin^−^) in cytokines which favour lymphopoiesis predominantly generates pro-B-cells, while overexpression of PU.1 in these cells dramatically skews commitment towards the myeloid lineage and results in a marked increase in macrophage production [2]. Therefore, high PU.1 expression promotes macrophage generation, while a lower level is important for B-cell development. Consistent with this, PU.1 levels have been estimated to be approximately eightfold higher in macrophages compared to B cells [2, 8]. 

PU.1 not only influences the myeloid-lymphoid decision of MPPs but also regulates the potential differentiation pathways to which CMPs and CLPs can commit. For instance, fine tuning of PU.1 levels in CMPs is important in directing differentiation towards MEPs or GMPs. PU.1 is expressed at high levels in GMPs and directs commitment towards the neutrophil and monocyte lineages. In contrast, PU.1 expression is downregulated in MEPs, and this is essential for the development of megakaryocytes and RBCs [7, 8, 20]. Within the lymphoid pathway, differentiation of CLPs to form B cells or T cells is accompanied by a decrease in PU.1 levels, and PU.1 is further silenced as T cells develop. Conversely, PU.1 expression in B cells gradually increases as they mature, although not to the extent observed in macrophages [2, 7, 8]. Together, these data collectively illustrate the importance of PU.1 in maintaining the HSC pool and in directing differentiation towards the myeloid and lymphoid pathways. The role of PU.1 in specific lineage differentiation programs will now be discussed in more detail.

## 3. PU.1 Is a Negative Regulator of Erythropoiesis

Downregulation of PU.1 expression in committed MEPs is important for erythroid differentiation. Low levels of PU.1 in early erythroid precursors are essential for them to undergo proliferation before terminally differentiating into RBCs. Using a PU.1 null mouse model, Back et al. showed that erythroid cells from these mice differentiate prematurely and are susceptible to apoptosis [6]. Another interesting transgenic model generated by Tavitian's group overexpresses PU.1 in all haematopoietic lineages except T cells [11]. This results in splenic hyperplasia and anaemia in approximately half of the transgenic mice, characterised by high numbers of immature blast cells and poorly haemoglobinised erythroblasts. The respective phenotypes from these two transgenic lines complement each other well. Whereas under normal physiological conditions PU.1 expression is diminished to allow terminal differentiation of erythroblasts, the persistent expression of PU.1 in Tavitian's mouse line results in a maturation block with an overrepresentation of immature erythroblasts [11, 20, 23]. Conversely, elimination of PU.1 results in premature differentiation of these cells. 

In fact, several groups have clearly demonstrated this point utilising the widely employed MEL (murine erythroleukemia) cell line. This cell line is derived from mice infected with Friend virus where the SFFV component has integrated into the Spi-1/PU.1 locus and transcriptionally activates the gene, resulting in erythroblast transformation [1, 24]. MEL cells, which exhibit ectopic PU.1 expression, are arrested at the erythroblast stage and undergo continual proliferation without differentiation [25, 26]. Treatment with chemical agents such as DMSO (dimethyl sulfoxide) or HMBA (hexamethylene bisacetamide) results in growth arrest, erythroid differentiation, and haemoglobin production, along with a concomitant decrease in PU.1 expression [27, 28]. The reduction in PU.1 expression is crucial for the differentiation of these cells. Forced expression of PU.1 in MELs inhibits DMSO- and HMBA-induced differentiation, while silencing PU.1 in these cells has been shown to be sufficient to drive terminal differentiation in the absence of any chemical inducers [25, 27–29].

PU.1 thus promotes erythroid cell proliferation and prevents differentiation. It has been proposed that one mechanism by which PU.1 does this is by regulating controllers of the cell cycle [25]. PU.1 directly activates expression of Cdk6 (cyclin-dependent kinase 6), a G1 phase-specific cell cycle kinase known to inhibit MEL cell differentiation [25, 30]. Cdk6 associates with D-cyclins to regulate cell cycle progression through the G1 phase, and like PU.1, Cdk6 is constitutively expressed in proliferating MELs [30]. After chemical induction, downregulation of PU.1 results in decreased Cdk6 levels, thus arresting proliferation and permitting differentiation. 

Another established mechanism by which PU.1 inhibits erythroid differentiation is by antagonising the erythroid regulator Gata-1. These two transcription factors are known to exert opposing effects on each other, and the interplay between these two master regulators is instrumental in the decision for CMPs to commit towards either the myeloid or erythroid lineage [31, 32]. PU.1 interacts with Gata-1 and prevents its transcriptional activity while conversely, Gata-1 inhibits PU.1 function by disrupting its interaction with the coactivator c-Jun [32–34]. Hence, these data collectively suggest that PU.1 inhibits erythroid differentiation by upregulating Cdk6 to promote proliferation and by antagonising the master erythroid regulator Gata-1. This may also explain why PU.1 expression must be downregulated during erythropoiesis to allow normal production of RBCs.

## 4. PU.1 Is a Master Myeloid Regulator

Both neutrophils and monocytes are generated from GMPs. Differentiation towards either of these cell fates is highly dependent on PU.1 [7, 8]. Multiple knockout models have demonstrated the importance of PU.1 in myelopoiesis; abrogation of PU.1 results in a marked lack of CMPs, defective granulocytic neutrophil production, and an absence of mature macrophages [4, 5, 12, 35]. Because PU.1 is absolutely required early during myelopoiesis at the CMP stage, questions have been asked as to whether it is dispensable once cells have committed to the CMP pathway. In answering this, Iwasaki et al. purified PU.1 null CMPs and GMPs from a conditional knockout mouse and assessed their capacity to form myeloid colonies in culture [12]. Both CMPs and GMPs were found to be unable to contribute to the mature myeloid fraction, indicating that PU.1 is further required following commitment to the myeloid lineage to promote differentiation to generate granulocytes and macrophages. PU.1 has since been shown to enable committed cells to respond to a variety of myeloid growth factors by regulating the expression of a number of myeloid-specific genes, including the cytokine receptors granulocyte/macrophage colony-stimulating factor receptor *α* (GM-CSFR*α*), granulocyte-CSFR (G-CSFR), macrophage-CSFR (M-CSFR), and interleukin-7 receptor *α* (IL-7R*α*) [36, 37]. 

### 4.1. Regulation of Macrophage versus Neutrophil Production

As a master myeloid regulator, PU.1 does not only regulate GMP development but also directs the differentiation pathways that give rise to both neutrophils and macrophages. Again, precise control of PU.1 levels in GMPs is required to direct these distinct differentiation programs. Different dosage of PU.1 expression in GMPs modulates distinct regulatory networks which involve a number of lineage-specific transcription factors such as Egr-2 (early growth response-2), Gfi-1 (growth factor independent-1), and C/ebp*α* (CCAAT enhancer-binding protein *α*) [21, 38, 39]. 

Using myeloid cell lines with an inducible transgene allowing high or low levels of PU.1, Laslo et al. revealed that low expression of PU.1 activates a mixed lineage of macrophage and neutrophil genes [39]. When PU.1 levels exceed a certain threshold, this induces the expression of Egr-2 and the transcriptional repressor Nab-2. Together, Egr-2 and Nab-2 repress the expression of Gfi-1, a transcription factor that promotes neutrophil differentiation. This results in the silencing of neutrophil genes and promotes macrophage differentiation [21, 39]. PU.1 thus indirectly represses Gfi-1 expression and conversely, PU.1 levels are elevated in Gfi-1 knockout mice [21]. Similar to Gata-1 and PU.1 antagonising each other's function in the myeloid-erythroid decision of CMPs, it appears that Gfi-1 and PU.1 negatively regulate each other's expression to determine the macrophage-neutrophil decision of GMPs [21]. 

Like Gfi-1, the neutrophil transcription factor C/ebp*α* is also expressed in myeloid cells and antagonises PU.1 to direct neutrophil differentiation. Dahl et al. showed that haematopoietic progenitors expressing high levels of inducible PU.1 predominantly develop into macrophages when cultured in IL-3 (interleukin-3) [38]. However, when these cells are pretreated with G-CSF (which promotes granulocyte development) prior to PU.1 induction, they display an upregulation of C/ebp*α* and instead form neutrophils. Thus, it can be deduced that with low levels of PU.1 in GMPs, the expression of Gfi-1 and C/ebp*α* are sufficient to antagonise PU.1 function by repressing macrophage genes and promoting neutrophil development. In contrast, when lineage commitment is directed towards macrophage development, PU.1 expression is upregulated to overcome the antagonism imposed by the two neutrophil regulators.

## 5. Regulation of Lymphopoiesis: PU.1 in B-Cell and T-Cell Maturation

In addition to being a major myeloid regulator, PU.1 has also been shown to modulate lymphopoiesis, the process that gives rise to B cells and T cells. PU.1 mutant mice exhibit a loss of the B and T cell compartments and develop fatal septicaemia within 2 days of birth owing to a lack of mature and functional immune cells [5, 35]. Studies investigating the pattern of PU.1 expression using GFP-reporter mice have revealed that PU.1 levels increase as B-cells mature, while PU.1 is completely silenced in mature T cells [7, 8]. Despite the requirement for PU.1 for B-cell production, its expression is dispensable once progenitors are committed to the lymphoid lineage as revealed by Iwasaki et al. [12]. PU.1 null CLPs generate B cells and express B-cell genes at comparable levels to wildtype CLPs in culture. *In vivo*, targeted deletion of PU.1 after the pre-B-cell stage does not disrupt immunoglobulin expression or the cells' response to a variety of mitogenic agents. This suggests that PU.1 is not essential for B-cell maturation once it has directed progenitors to the CLP stage. One possible explanation for this is that other members of the ETS family of transcription factors, such as Spi-B, may have functional redundancy with PU.1 in B cells [40, 41]. Similar to in B cells, PU.1 is only required at the early CLP stage for T-cell generation. Once cells develop to pro-T-cells, PU.1 expression is dramatically silenced. Silencing of PU.1 is required for T cell maturation and in fact, forced overexpression of PU.1 results in growth arrest and a T-cell maturation block [42]. 

## 6. Dosage-Dependent Regulation and Pathogenesis

It is clear that precise levels of PU.1 expressed at different stages within the haematopoietic lineage are crucial in directing proper differentiation and cell fate commitment (Figure 1). Hypomorphic mouse lines expressing varied doses of PU.1 have been particularly helpful in providing a better picture on how PU.1 activates and represses specific sets of genes. The PU.1^BN^ and PU.1^Blac^ mice have been estimated to express approximately 20% (“high” concentration) and 2% (“low” concentration) of PU.1 compared to wildtype mice, respectively, [22, 35]. By performing microarrays on PU.1^BN^, PU.1^Blac^, and PU.1^−/−^myeloid cell lines, it was revealed that PU.1 can regulate its targets in four distinct modes [22]. Some target genes are activated or repressed equally at both “high” and “low” concentrations of PU.1; others are exclusively activated or repressed at either “high” or “low” levels of PU.1 but not both, and the final group of targets can be activated or repressed in a dose-dependent manner by PU.1, that is, the degree of their transcriptional regulation is dependent on the level of PU.1. Targets which are repressed in such a “gradient” manner include erythroid and T-cell-specific genes, while genes which are activated include the myeloid genes. This illustrates the dependence on PU.1 levels in the regulation of these lineage-specific genes and may in part explain why PU.1 expression varies in certain cell types during specific stages of haematopoiesis. 

In fact, when this delicate expression control is disrupted, the balance between different haematopoietic regulators is upset, and in many occasions, this results in oncogenic transformation [11, 33, 43]. A notable example is the SFFV-induced transcriptional activation of PU.1 in Friend virus infected mice which develop acute erythroleukaemia [1]. In this instance, the high level of PU.1 expression disrupts the stoichiometry between PU.1 and Gata-1. Zhang et al. showed by EMSA (electrophoretic mobility shift assays) that PU.1 directly interacts with the Gata-1 DNA recognition motif thereby blocking its DNA-binding activity [33, 34]. Gata-1 transactivation which ordinarily promotes normal erythroid differentiation is consequently inhibited and allows erythroleukaemic transformation [33, 44]. Studies have also revealed that deletion of a −14 kb upstream regulatory region (URE) reduces PU.1 expression by 80% in mice and results in acute myeloid leukaemia (AML) and T-cell lymphoma [10, 13]. These mice have an accumulation of immature myeloblasts and neutrophils in both the bone marrow and spleen accompanied by an increased number of immature white blood cells in peripheral blood. Persistent PU.1 expression also induces transformation of early T-cell progenitors, which subsequently develop into aggressive lymphomas [10]. Lastly, a series of PU.1 mutations have been shown to associate with AML in humans [43]. These mutations are thought to abrogate PU.1 function by a number of mechanisms, for example, by reducing its DNA-binding capacity or by disrupting its interaction with coregulators or other transcription factors. However, attempts by a number of other groups have failed to demonstrate an association between PU.1 mutations and AML [45–47], suggesting that the mutations identified in the Mueller study may be linked to rare subsets of the disease. Nonetheless, this does not exclude the possibility that PU.1 haploinsufficiency can promote leukaemogenesis in humans, further emphasising the importance of maintaining precise PU.1 expression levels in haematopoietic cells.

## 7. Conclusion

Through the study of a variety of transgenic mouse models and cell lines, PU.1 has emerged as a key regulator of haematopoiesis. The expression of PU.1 is exquisitely and dynamically controlled throughout the various haematopoietic differentiation pathways. Precise PU.1 levels direct cell fate by regulating gene expression in a graded manner and by antagonising the function of other haematopoietic regulators, such as Gata-1. Disturbing the normal expression pattern of PU.1 in haematopoietic cells can lead to skewed lineage commitment and in some instances, oncogenesis. Indeed, PU.1 haploinsufficiency has been linked with AML in humans. Understanding the mechanisms by which PU.1 and other factors regulate haematopoietic differentiation is of significant importance, as manipulation of these factors may offer a therapeutic option for the treatment of leukaemias and other haematopoietic disorders.
